# Hematemesis Following Appendicectomy

**Published:** 1903-09

**Authors:** 


### HEMATEMESIS FOLLOWING APPENDICECTOMY.

George Ryerson Fowler, (*Brooklyn Med. Jour.*, July, 1903,) reports a fatal case. The occurrence of gastric hemorrhage has added another to the already long list of grave sequelæ of appendicitis. Whether this hemorrhage depends upon the toxic effects of septic bacterial products which enter the circulation and produce necrosis of the cells of the gastric mucosa, this in its turn being followed by ulceration due to the digestive action of the gastric juice, as claimed by Nitsche, or upon embolism of the vessels of the gastric mucosa, as shown in the case herewith reported, matters but little from the clinical standpoint. In either case the toxicity of the appendical lesion is responsible for the fatal issue which in the vast majority of cases ensues.

